# Breast cancer suppression by aplysin is associated with inhibition of PI3K/AKT/FOXO3a pathway

**DOI:** 10.18632/oncotarget.19209

**Published:** 2017-07-12

**Authors:** Xinling Zhang, Tingting Zhuang, Zhengyan Liang, Li Li, Meilan Xue, Jia Liu, Hui Liang

**Affiliations:** ^1^ The Institute of Human Nutrition, Medical College of Qingdao University, Qingdao 266021, China; ^2^ Key Laboratory of Marine Drugs, Chinese Ministry of Education, Key Laboratory of Glycoscience & Glycotechnology of Shandong Province, School of Medicine and Pharmacy, Ocean University of China, Qingdao 266003, China

**Keywords:** aplysin, PI3K/AKT, cell proliferation, apoptosis, breast cancer

## Abstract

Aplysin, a bromosesquiterpene isolated from Aplysia kurodai, was explored as a potential anti-breast cancer agent by us. However, the mechanisms underlying the anticarcinogenic effect of aplysin remain unclear. Here, aplysin was found to remarkably suppress tumor growth *in vivo*, inhibit cell proliferation and promote apoptosis *in vitro*. Additionally, we demonstrated that aplysin attained these effects in part by down-regulating PI3K/AKT/FOXO3a signaling pathway. Aplysin treatment inhibited the phosphorylation levels of AKT (Ser-473) and AKT-dependent phosphorylation of FOXO3a (Ser-253) in breast cancer cell lines and breast cancer tissues. The expression levels of FOXO3a-targeted genes were also destabilized by aplysin, cyclin D1 and Bcl-XL were declined; however, p21^CIP1^, p27^KIP1^, Bim, TRAIL and FasL were increased both *in vivo* and *in vitro*. Furthermore, activation of the PI3K/AKT signaling pathway by an activator and silencing of FOXO3a by shRNA protected the cells from aplysin mediated growth suppression and apoptosis. In summary, our findings revealed that aplysin could suppress breast cancer progression by inhibiting PI3K/AKT/FOXO3a pathway, thereby suggesting a potential role of aplysin as a chemoprevention drug for patients with breast cancer.

## INTRODUCTION

Breast cancer is the most frequently diagnosed cancer and the leading cause of cancer death in women worldwide [1]. Although the precise mechanism which causes breast cancer is not fully established, accumulating evidence indicates that Phosphatidylinositol-3-kinase (PI3K)/AKT pathway is involved in tumorigenesis and progression of various cancer types including breast cancer [2–4]. As much as 70% of breast cancers are associated with a hyperactive PI3K/AKT pathway [4, 5] and activated AKT modulates the function of numerous substrates involved in the regulation of cell survival, cell cycle progression and cellular growth [6]. PI3K/AKT signalling pathway plays a major role not only in tumor development but also in the tumor's potential response to cancer treatment [6]. Therefore, many of the new “targeted agents” have been specifically designed to act on PI3K/AKT-related targets. FOXO3a, a member of the Forkhead box O (FoxO) transcription factor family, functions downstream of PI3K/AKT pathway and acts as a tumor suppressor in the majority of human cancers [7, 8]. In fact, FOXO3a has served as the target of several cancer drugs. For example, chemotherapeutic drugs paclitaxel and KP372−1, which are currently used in the treatment of breast carcinoma and acutemyeloid leukemia, can activate FOXO3a by reducing AKT activity [9, 10]; gene-targeted drugs such as trastuzumab and cetuximab enhance the drug sensitivity of drug-resistant tumor cells by activating FOXO3a activity and thus causing overexpression of FOXO3a responsive genes such as Bim, p21^CIP1^ and p27^KIP1^ [8, 11, 12]. Thus, increasing FOXO3a activity has become an important cancer therapeutic strate.

**Figure 1:**
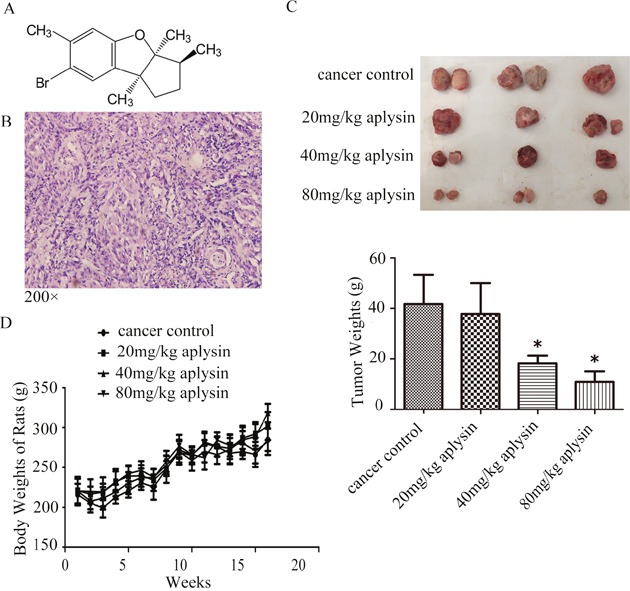
Aplysin inhibited the growth of breast tumors *in vivo* The structure of aplysin was showed here **(A)**. Female Wistar rats were adaptively fed for one week before starting the experiment. 100 mg/(kg·BW) DMBA was injected subcutaneously into each animal. After three days, all rats were randomly divided into four groups with fifteen rats in each group. Treated group respectively received 20, 40 and 80 mg/kg body weight aplysin by oral gavage everyday while control group received vehicle alone. All breast carcinomas were confirmed by histopathological analysis **(B)**. Effect of aplysin on tumor weight **(C)** and the rat body weights **(D)** were evaluated. Values in C and D are means ± SEM of 15 samples.* P<0.05 represents statistically significant when compared with control.

To date, although the therapeutic effects of breast cancer are markedly increased, it is still the leading cause of cancer death in women. Therefore, it is urgent to explore new drugs with good antitumor activities and no additional side effects to prevent and treat breast cancer. According to traditional Chinese medicine record, aplysia is useful to alleviate the symptoms of many kinds of inflammations and has antitumor activity. Literatures reported that three compounds Aplysianin-A [13], Aplysianin-E [14] and Aplysianin-P [15] extracted from Aplysia kurodai have convincing anti-tumor activities. Aplysin (C15H19OBr), another extract from Aplysia kurodai, is a bromosesquiterpene with a molecular weight of 295 [16] (Figure 1A). It is isolated by Japanese scholars S. Yamamura and Hiratain in 1963, earlier than that of the above three Aplysia kurodai extracts but its biological activity has not been explored until our laboratory finds its anti-tumor effects [17, 18].

In the present study, our results demonstrated that orally feeding aplysin significantly suppressed the growth of 7, 12-dimethylbenz[a]anthracene (DMBA) -induced breast tumor tissues in rat models. Additionally, cell proliferation was inhibited and apoptosis was induced by aplysin in human breast cancer cells. Tumor inhibition by aplysin was realized by inhibition of PI3K/AKT/FOXO3a pathway. Consistent with this observation, PI3K/AKT activator treatment and silencing of FOXO3a by shRNA *in vitro* both rescued aplysin mediated growth suppression and apoptosis.

## RESULTS

### Aplysin inhibited tumor growth in rat models

To test the possibility that aplysin preventive treatment would suppress breast tumor growth, female Wistar rats with mammary gland tumor induced by DMBA were fed 0, 20, 40 and 80 mg/kg aplysin each day respectively for 16 weeks, and tumor growth was periodically recorded. 8 weeks after DMBA injection, 12 rats of Cancer Control group presented at least 1 tumor (80%). 13 weeks later, all of them (100%) developed tumors. Histopathological analyses were performed on mammary tissues from all DMBA-treated animals and the carcinomas exhibited solid patterns combined with comedo-necrosis predominating, peripheral inflammatory cell infiltration and dense tumor cells. Moreover, the nuclear was large, deep-stained, nucleocytoplasm ratio was increased and several dark mitotic figures were seen (Figure 1B). The results showed that oral gavage of 40 and 80 mg/kg aplysin significantly reduced the tumor growth. The tumor weights dissected from 40 and 80 mg/kg aplysin treated rats were about 56% and 74% less than that of control rats (Figure 1C). The body weights of the rats did not changed significantly, indicating no apparent systemic toxicity in aplysin-treated rats (Figure 1D).

### Aplysin suppressed cell proliferation and induced apoptosis in breast cancer cells

**Figure 2:**
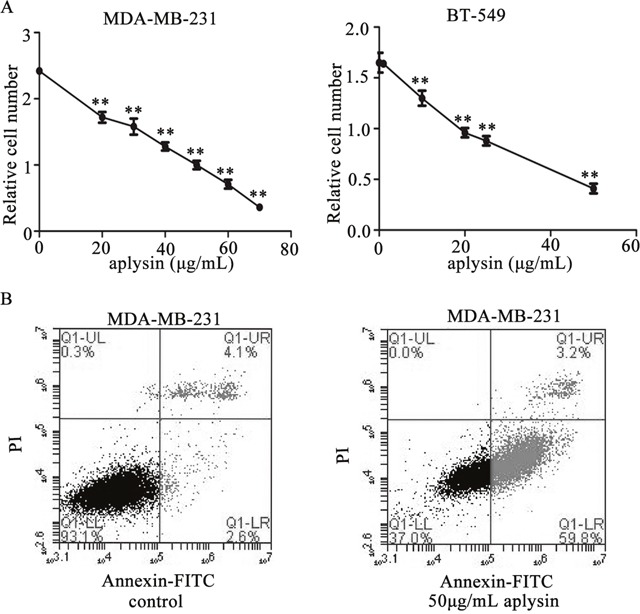
Aplysin suppressed cell proliferation and induced apoptosis *in vitro* MDA-MB-231 and BT-549 cells were treated with varying concentrations of aplysin for 48 h and the ability of cell survival was measured by MTT assay **(A)**. MDA-MB-231 cells were treated with 50 μg/mL aplysin for 36 h and apoptosis was evaluated using Annexin V-FITC kit. The lower right quadrant (annexin V+/PI−) represents early apoptosis, and the upper right quadrant (annexin V+/PI+) represents late apoptosis and necrosis **(B)**. Each bar represents means ± SEM of at least three independent experiments. ** P<0.01 represents statistically significant when compared with control.

To detect the effect of aplysin on breast cancer *in vitro*, 2 different breast carcinoma-derived cell lines, MDA-MB-231 and BT-549, were treated with aplysin. The MTT assay showed that significant reductions (more than 30%) was observed in proliferations of MDA-MB-231 and BT-549 cells when they are exposed to 20μg/mL aplysin or a higher dose for 48h (Figure 2A). And the half-maximal inhibitory concentration (IC50) of aplysin was 42.7 μg/mL in MDA-MB-231 cells and 25.2 μg/mL in BT-549 cells. Annexin-V/FITC analysis was performed by flow cytometry to characterize the early apoptosis of the breast cancer cells. As shown in Figure 2B, the lower right quadrant (annexin V+/PI−) represents early apoptosis, and the early apoptotic rates of MDA-MB-231 cells treated with 50 μg/mL of aplysin for 36 hours were 59.8±1.17% and significantly higher than that in control cells(≦3%) (P<0.01). These results had given an indication that aplysin suppressed cell proliferation and induced apoptosis in breast cancer cells.

### Tumor suppression of aplysin was achieved through inhibition of PI3K/AKT/FOXO3a pathway *in vivo*

**Figure 3:**
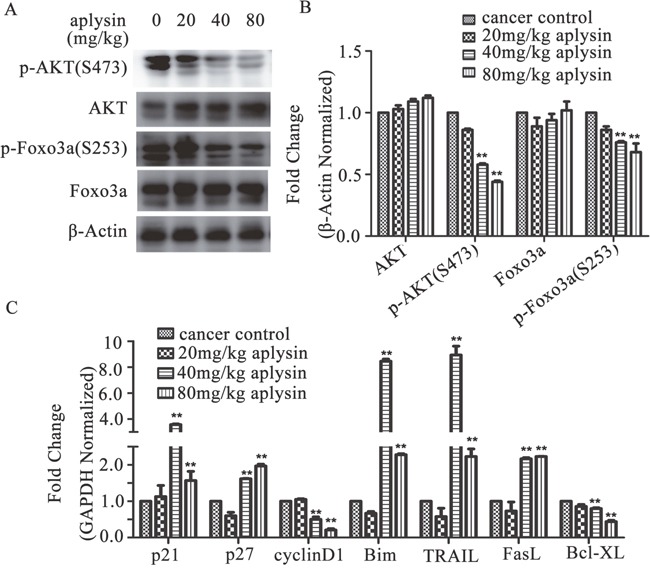
Breast cancer suppression of aplysin *in vivo* was ralated to the inhibition of PI3K/AKT/FOXO3a pathway The mechanism of tumor growth inhibition by aplysin was determined in the tumor lysates from control and aplysin-treated rats. Tumors were homogenized, lysed and 40 μg proteins were used for western blotting and p-AKT (Ser-473), AKT, p-FOXO3a (Ser-253), FOXO3a were detected and normalized to β-Actin housekeeping gene **(A** and **B)**. Tumors were also lysed to use for qPCR analysis and the mRNA levels of FOXO3a-regulated genes such as cyclinD1, p21, p27, Bim, TRAIL, FasL and Bcl-XL were checked and normalized to GAPDH housekeeping gene **(C)**. Valuesare means ± SEM of 15 samples. ** P<0.01 represents statistically significant when compared with control.

As reported, PI3K/AKT is constitutively activated in majority of breast tumors. We hypothesized that the tumor inhibition by aplysin in rat breast cancer models was due to inhibition of PI3K/AKT pathway. To test our hypothesis, constitutive protein and phosphorylation levels of AKT were examined in the tumor lysates by western blotting. As shown in Figure 3A and 3B, phosphorylation of AKT at Ser-473 was dratically suppressed by 40 and 80 mg/kg aplysin. However, there was no variation in the expression of AKT (Figure 3A and 3B). Next we investigated FOXO3a, which is an important downstream molecule of AKT pathway and a tumor suppressor in the human breast carcinoma. The results showed that the phosphorylated levels, but not protein levels of FOXO3a were decreased in 40 and 80 mg/kg aplysin-treated tumors (Figure 3A and 3B). Furthermore, the mRNA levels of FOXO3a-regulated genes were detected. Proliferation-associated genes cyclinD1 was dramatically declined; however p21^CIP1^ and p27^KIP1^ were visibly induced by aplysin. The pro-apoptotic proteins Bim, TRAIL and FasL were substantially increased and anti-apoptotic protein Bcl-XL was decreased in aplysin-treated tumors as compared to control tumors (Figure 3C). These results indicated that aplysin mediated breast tumor suppression by inhibiting PI3K/AKT/FOXO3a pathway.

### Aplysin down-regulated PI3K/AKT/FOXO3a pathway *in vitro*

**Figure 4:**
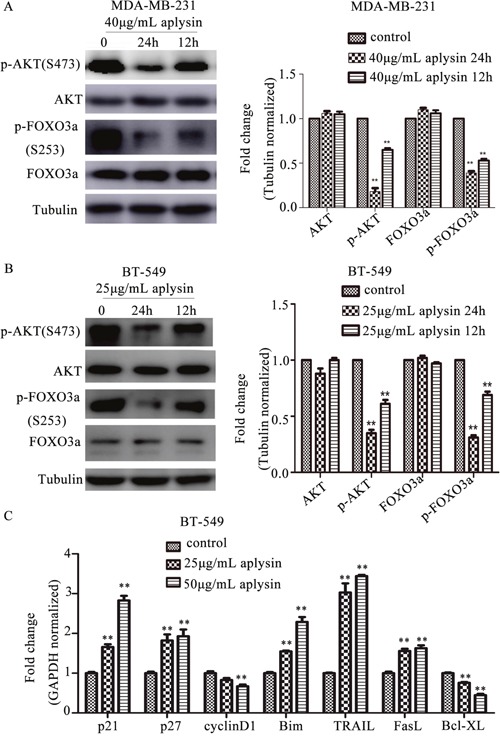
Aplysin inhibited breast cancer *in vitro* through suppressing PI3K/AKT/FOXO3a pathway Effect of aplysin on PI3K/AKT/FOXO3a pathway in MDA-MB-231 **(A)** and BT-549 **(B)** cells was detected. Cells were treated with 40 and 25 μg/mL aplysin respectively for 12 h and 24 h and immunoblotted for p-AKT (Ser-473), AKT, p-FOXO3a (Ser-253) and FOXO3a. The same blot was stripped and reprobed for Tubulin to ensure equal proteins loading. Bar diagram showed the quantitation of respective western blots. BT-549 cells which treated with 25 and 50 μg/mL aplysin respectively for 24 h were also subjected to real-time RT-PCR analysis and the mRNA level of cyclinD1, p21, p27, Bim, TRAIL and FasL was showed **(C)**. Each bar represents means ± SEM of at least three independent experiments.

To model and elucidate the molecular observations made *in vivo*, we treated MDA-MB-231 and BT-549 cells with 40 μg/mL and 25 μg/mL aplysin respectively for 12 h and 24 h. As shown in Figure 4A and 4B, aplysin treatment significantly suppressed the phosphorylation of AKT (Ser-473) and FOXO3a (Ser-253) both in MDA-MB-231 and BT-549 cells in a time-dependent manner. The protein levels of AKT and FOXO3a remained unchanged even after 24h treatment with aplysin. The results indicated that aplysin specifically targeted the activation of PI3K/AKT/FOXO3a pathway. Aplysin-induced FOXO3a activation, in turn, decreased the expression of cyclinD1 and Bcl-XL, and increased the levels of p21^CIP1^, p27^KIP1^, Bim, TRAIL and FasL. These results were consistent with what we obtained *in vivo* (Figure 4C).

### IGF-1 treatment rescued aplysin-mediated cell proliferation suppression and apoptosis

**Figure 5:**
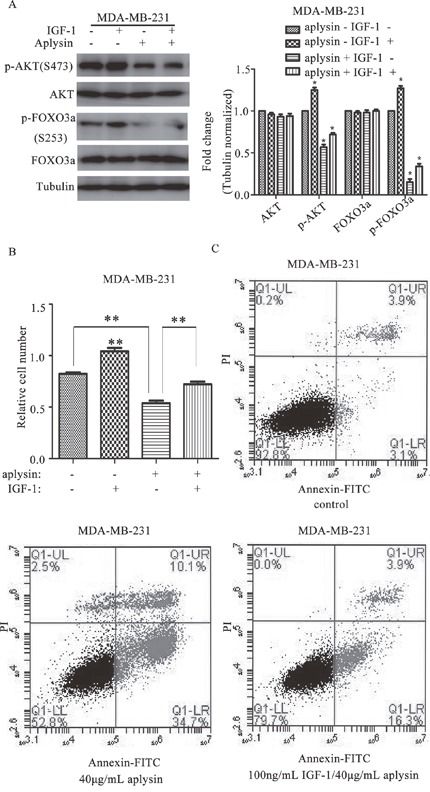
PI3K/AKT activation rescued aplysin mediated growth suppression and apoptosis Before being treated with 40 μg/mL aplysin, MDA-MB-231 cells were treated with 100 ng/mL IGF-1 for 1 h. And the level of p-AKT (Ser-473), AKT, p-FOXO3a (Ser-253) and FOXO3a were determined after aplysin treated for 24 h **(A)**. MDA-MB-231 cells were treated with 100 ng/mL IGF-1, 1 h later, 30 μg/mL aplysin was also added to the samples and the cell proliferation was measured by MTT assay after aplysin treated for 48 h **(B)**. MDA-MB-231 cells were treated with 100 ng/mL IGF-1 for an hour, and 40 μg/mL aplysin was added to and treated the cells for 36 h before subjected to Annexin-V/FITC analysis **(C)**. Each bar represents means ± SEM of at least three independent experiments.

To confirm the role of PI3K/AKT signaling in aplysin-mediated breast cancer suppression, breast cancer cells were treated with 100 ng/mL IGF-1, an activator of PI3K/AKT signaling. The results showed that the decline in AKT and FOXO3a phosphorylation by aplysin treatment was markedly recovered by IGF-1 in MDA-MB-231 cells (Figure 5A). Moreover, IGF-1 treatment also significantly recovered the viability of MDA-MB-231 cells inhibited by aplysin. The survival of MDA-MB-231 cells which was treated by 30 μg/mL aplysin was 65.4±1.2% whereas in IGF-1 and aplysin co-treated cells the survival was 87.8±1.6% indicating a 22.4% survival advantage (Figure 5B). We next determined apoptosis in control and IGF-1 treated cells after aplysin treatment by AnnexinV-FITC. The results showed that 40 μg/mL aplysin induced about 34.7±1.34% apoptosis whereas, in IGF-1 and aplysin co-treated cells, apoptosis was reduced to 16.3±0.54%, indicating 18.4% decrease (Figure 5C). Taken together, these results establish the critical role of AKT inhibition in aplysin-induced apoptosis and proliferation inhibition through FOXO3a in our model.

### FOXO3a-deficiency resulted in reversal of aplysin-mediated cell proliferation suppression and apoptosis

**Figure 6:**
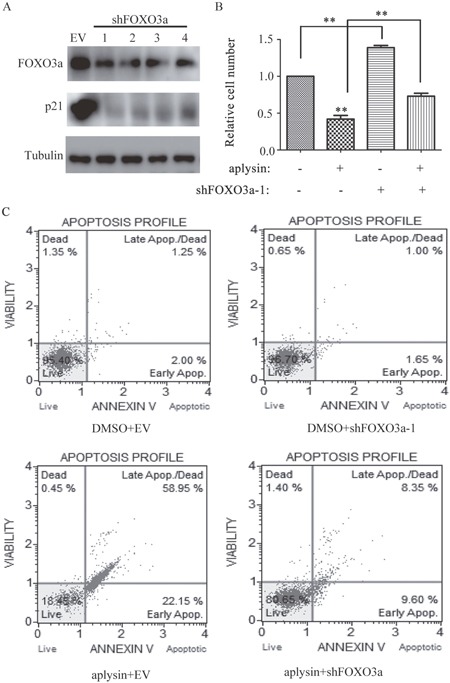
Knockdown of FOXO3a abrogated aplysin-mediated cell proliferation suppression and apoptosis BT-549 cells were transfected with shFOXO3a, the cell lysates of each group were prepared and probed for FOXO3a and its target gene p21 by western blot **(A)**. BT-549 cells were transfected with shFOXO3a, and then further incubated in the presence of aplysin for 48 or 36 h, the survival **(B)** and apoptosis **(C)** of cells were detected respectively. Each bar represents means ± SEM of at least three independent experiments. EV, empty vector; DMSO, the solvent of aplysin.

To determine whether the anti-tumor effects of aplysin depended on FOXO3a, cells were transfected with four LV3 lentiviral constructs expressing a short hairpin targeting FOXO3a (shFOXO3a-1, shFOXO3a-2, shFOXO3a-3 and shFOXO3a-4), LV3 was used as empty vector control. The targeting FOXO3a sequences were listed in Supplmentary Table 1. The expressions of FOXO3a in BT-549 cells were dramatically suppressed by all of shFOXO3as (Figure 6A). Moreover, as expected, FOXO3a knockdown consequently suppressed the protein expressions of p21^CIP1^(Figure 6A and 6B). FOXO3a silencing resulted in marked attenuation of inhibition of BT-549 cell proliferation by aplysin (Figure 6B). Annexin-FITC Apoptosis assay also showed that the combination of shFOXO3 and aplysin decreased BT-549 cell apoptosis compared with cells treated with aplysin alone (Figure 6C). These data indicated that the reduction of FOXO3a expression weakened apoptosis and the attenuation of cell proliferation by aplysin.

## DISCUSSION

**Figure 7:**
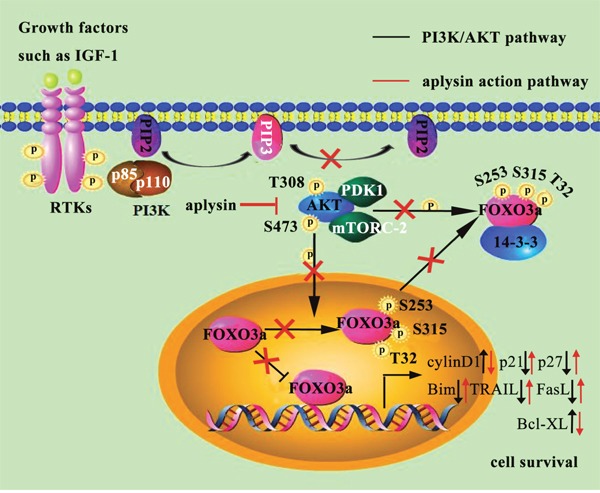
Molecular mechanisms underlying the anticancer effects of aplysin in regulating human breast cancer growth

We had previously explored that aplysin suppressed cell proliferation of breast cancer. However, the exact mechanism was not clear. This study demonstrated that aplysin suppressed tumor growth *in vivo* and also decreased cell proliferation and increased apoptosis *in vitro*. Tumor suppression by aplysin was verified to link with the inhibition of PI3K/AKT/FOXO3a pathway. And the inhibition of PI3K/AKT/FOXO3a caused down-regulation of cyclinD1 and Bcl-XL and up-regulation of p21^CIP1^, p27^KIP1^, Bim, TRAIL and FasL, finally achieving inhibition of cell proliferation and promotion of apoptosis. In conclusion, our results demonstrated that aplysin mediated breast tumor suppression by inhibiting PI3K/AKT/FOXO3a pathway (Figure 7).

Constitutive activation of AKT has been reported in various cancer types [19] and over 55% of the cancers have hyper-activation of AKT, making it as an attractive molecular target [20]. Some studies suggested that the activation of PI3K/AKT pathway could enhance resistance to chemotherapy [21–23] and molecule-targeted treatment [24–26]. Here aplysin substantially suppressed the phosphorylation of AKT *in vivo* and *in vitro*, indicating that aplysin suppressed breast cancer by targeting constitutively activated-AKT.

FOXO3a regulates cell survival, cell death, cell metabolism and resistance [27–30] and FOXO3a overexpression inhibits breast cancer growth *in vitro* and *in vivo* [31, 32]. AKT-dependent phosphorylation of FOXO3a leads to its cytoplasmic retention by 14-3-3 proteins and loss of target gene activation [33–35]. Thus, signaling through the PI3K/AKT cascade, by inactivating FOXO3a, results in decreased negative cell-cycle regulators, such as p21^CIP1^ [36] and p27^KIP1^ [37] and increased positive cell-cycle regulators, such as cyclinD1 [38]. AKT-mediated phosphorylation of FOXO3a also promotes cell survival throught inhibiting pro-apoptotic proteins such as TRAIL [39], Bim [40], FasL [41] and promoting anti-apoptosis protein such as Bcl-XL [42]. Our results revealed that the phosphorylation at Ser-253 but not protein level of FOXO3a was decreased with aplysin treatment. Furthermore, FOXO3a responsive genes cyclinD1 and Bcl-XL were decreased while the others were all up-regulated by aplysin. Activation of PI3K/AKT pathway and silencing FOXO3a severely abrogated the apoptosis-inducing and growth suppressive effects of aplysin. These results provided the evidence that aplysin induced apoptosis and inhibited cell proliferation by inhibiting PI3K/AKT/FOXO3a pathway.

In conclusion, our *in vitro* and *in vivo* results demonstrated that aplysin suppressed the growth of the breast tumor in strong association with an inhibition and targeting of PI3K/AKT, and probably in major part, their downstream FOXO3a pathway. These studies provided a foundation upon which to further examine the role of aplysin in cancer therapy, particularly applicable in diseases where PI3K/AKT is known to be the oncogenic driver, including breast, colon, ovarian, prostate and pancreatic cancers.

## MATERIALS AND METHODS

### Animals

Female Wistar rats [200±5g], aged 7 weeks were obtained from Qingdao Laboratory Animal Center and housed in a room maintained at constant temperature [22 ± 2°C] and humidity [55 ± 5%] with 12 h of light and 12 h of darkness each day. The rats were acclimated to the environment for one week prior to the initiation of the experiment. All of the procedures involving rats were conducted in strict compliance with relevant laws, the Animal Welfare Act, the Public Health Services Policy, and the guidelines established by the Institutional Animal Care and Use Committee of the University.

### Experimental design

Aplysin was purified from the red alga by our laboratory and the purity is 97.6%. To assess the antitumor efficacy of aplysin and the mechanism *in vivo*, a single dose of 100 mg/kg body weight (BW) DMBA (Sigma-Aldrich, St. Louis, Missouri, USA; Cat.# D3254) was injected subcutaneously into Female Wistar rats [8 weeks old]. Three days later, the rats were divided according to BW, which were similar, into four equal groups of fifteen animals each. Group 1: Cancer Control, the rats were given equal volume of soybean oil by gavage once a day. Group 2: Low-dose aplysin intervention, aplysin was given in the dose of 20 mg/kg body weight in soybean oil every day. Group 3: Mid-dose aplysin intervention, aplysin was given in the dose of 40 mg/kg body weight in soybean oil every day. Group 4: High-dose aplysin intervention, aplysin was given in the dose of 80 mg/kg body weight in soybean oil every day. Animals were observed daily, and all the necessary data were recorded. The experiment was terminated at the end of the 16^th^ week. All animals were sacrified by cervical dislocation after an overnight fast. Normaland suspicious lesions were rapidly removed, measured, and rinsed in physiological saline.

### Histological analysis

Tissue specimens from the mammary gland of female rats of several experimental groups were collected and fixed in 10% formalin, processed, and embedded into paraffin blocks. Sections were cut at 5 μm thickness and stained with hematoxylin and eosin. The slides were examined under a light microscope (Olympus BX51) to observe for adenocarcinoma and lymph node, and confirmed by an experienced histopathologist.

### Cell culture

Human breast cancer cell line MDA-MB-231 and BT-549 were obtained from the Cell Bank, China Academy of Sciences (Shanghai, China). MDA-MB-231 cell was maintained in L15 medium (HyClone; Fisher Scientific, Mississauga, ON, Canada; Cat.#SH30525.01), BT-549 was maintained in RPMI-1640 medium (Gibco-Invitrogen, Carlsbad, CA, USA; Cat.# 31800022) with 100 ng/mL cholera toxin (Sigma-Aldrich, St. Louis, Missouri, USA; Cat.# C8052). The base medium should be supplemented with 10% fetal bovine serum (FBS; Gibco-Invitrogen, Carlsbad, CA, USA; Cat.#16000-044) and penicillin/streptomand (Invitrogen, Cat.# 15140–122) at 37°C in a humidified incubator with 5% CO2.

### Western blot analysis

MDA-MB-231 and BT-549 cells were treated with 40μg/mL and 25μg/mL aplysin respectively for 12h and 24h. Cell lysates were used for WB analysis. Antibodies to AKT (CellSignaling Technology, Beverly, MA; Cat.#2920), phospho-AKT (S473) (Cell Signaling Technology, Beverly, MA; Cat.#4060), FOXO3a (Cell Signaling Technology, Beverly, MA; Cat.#2497), phospho-FOXO3a (S253) (Cell Signaling Technology, Beverly, MA; Cat.#9466) and Tubulin (Santa Cruz, CA, USA; Cat.#sc-32292) and β-Actin (Santa Cruz, CA, USA; Cat.#sc-47778) were used for detection. Forty microgram protein was subjected to SDS-PAGE and western blot was carried out as described previously [43].

### RNA isolation and quantitative real-time PCR

Total RNA extraction, reverse transcription, and Quantitative PCR assays were carried out as previously described [43]. RNA extraction from tissue samples used the Animal Tissue RNA Purification Kit (TIANGEN Biotech, Beijiing, China; Cat.#DP431) according to the manufacturer's protocol. RNA extraction from cell samples used the Total RNA Extraction Kit (BioTeke, Beijiing, China; Cat.#RP1202) according to the manufacturer's protocol. The oligonucleotides used as PCR primers were listed in Supplementary Table 2.

### Cell proliferation assay

3-(4, 5-dimethyl-2-thiazolyl)-2, 5-diphenyl-2-H-tetrazolium bromide (MTT) is available from Sigma-Aldrich of St. Louis, Mo. as Cat. No. M2128. To detect the inhibitory effect of aplysin on cell viability, approximately 1×10^4^ MDA-MB-231 and BT-549 cells were seeded on each well of the 96-well plates respectively. After 24 h, cells were treated with varying concentrations of aplysin, 48 h later, the MTT assay was used for cell proliferation assay. To confirm the involvement of PI3K/AKT pathway in aplysin mediated cell proliferation inhibition, MDA-MB-231 cells were pre-treated with 100 ng/mL IGF-1(R&D systems, Abingdon, UK; Cat.#291-G1) for 1 h before 40 μg/mL aplysin was added, and the cell proliferation ablility was detected 48h later.

### Annexin-FITC apoptosis assay

MDA-MB-231 cells were treated with 50 μg/mL aplysin for 36 h and apoptosis was evaluated using Annexin V-FITC kit (BD Bioscience Pharmingen^™^, San Deigo, CA; Cat.# 556547) by flow-cytometer (Accuri C6, MI) according to manufacturer's instructions. To verify the role of PI3K/AKT pathway in aplysin-mediated apoptosis, MDA-MB-231 cells was treated with 100 ng/mL IGF-1, 1 hour later, 40 μg/mL aplysin was added to the cells and apoptosis was evaluated 36 h later.

### Statistical analysis

Data were analyzed by Students’ test using the SPSS 23.0 software program, and P<0.05 was considered statistically significant. Data were presented as the means ±standard error of measurement (SEM) of at least three independent experiments.

## SUPPLEMENTARY TABLES


