# Supplementary material for: Breast cancer suppression by aplysin is associated with inhibition of PI3K/AKT/FOXO3a pathway
Source: Oncotarget. 2017 Jul 12;8(38):63923–34. doi: 10.18632/oncotarget.19209 (PMC5609973; doi:10.18632/oncotarget.19209)
Supplement: Supplementary file 1 [file oncotarget-08-63923-s001.pdf]

Breast cancer suppression by aplysin is associated with inhibition of PI3K/AKT/FOXO3a pathway

SUPPLEMENTARY MATERIALS

Supplmentary Table 1: shFOXO3a targeting sequences

shFOXO3a	sequences
shFOXO3a-1	5'-GCACAACCTGTCACTGCATAG-3'
shFOXO3a-2	5'-GGGCTGTCTCCATGGACAATA-3'
shFOXO3a-3	5'-GGAACGTGATGCTTCGCAATG-3'
shFOXO3a-4	5'-GCTCACTTCGGACTCACTTAG-3'

Supplmentary Table 2: Primers for Quantitative real-time PCR (5'→3')

**The oligonucleotides used as PCR primers**	
cyclinD1(rat) forward: GAGGCGGATGAGAACAAGCA	cyclinD1(homo) forward: CCGATGCCAACCTCCTCAAC
cyclinD1 (rat) reverse: CGGTAGCAGGAGAGGAAGTTG	cyclinD1 (homo) reverse: CTCCTCCTCGCACTTCTGTTC
p21 (rat) forward: AAGCAGTCACAGCCTAGAACAA	p21 (homo) forward: GTCCAGCGACCTTCCTCATC
p21 (rat) reverse: GAGAGCAGCAGATCACCAGATT	p21 (homo) reverse: TAGCCTCTACTGCCACCATCT
p27(rat) forward: GAAGCACTGCCGAGATATGGA	p27(homo) forward: GAGTGGCAAGAGGTGGAGAAG
p27 (rat) reverse: CACCTCCTGCCACTCGTATC	p27 (homo) reverse: ATGCGTGTCCTCAGAGTTAGC
Bim (rat) forward: CCAGAGATACGGATCGCACAG	Bim (homo) forward: GTATTCGGTTCGCTGCGTTCC
Bim (rat) reverse: CTCCATACCAGACGGAAGATGA	Bim (homo) reverse: TCTACCTTCTCGGTCACACTCA
TRAIL (rat) forward: AATCGGACTAGCTTGCTTCTCA	TRAIL (homo) forward: GGCTGCCTGGCTGACTTACA
TRAIL (rat) reverse: GCTGATACAGTTGCCTCTTGAC	TRAIL (homo) reverse: GGAGCACTGTGAAGATCACGATC
FasL (rat) forward: TCCACCACCACCTCCATCAC	FasL (homo) forward: TGGTTCTGGTTGCCTTGGTAG
FasL (rat) reverse: CATTCCAACCAGAGCCACCAG	FasL (homo) reverse: CCTTGAGTTGGACTTGCCTGT
GAPDH (rat) forward: AGTTCAACGGCACAGTCAAGG	GAPDH (homo) forward: TGATGGTCGGCTGCGTGTA
GAPDH (rat) reverse: ACATACTCAGCACCAGCATCAC	GAPDH (homo) reverse: GGGCTCAGGGCATAACAGAA
